# Biomimetic Composite Scaffold With Phosphoserine Signaling for Bone Tissue Engineering Application

**DOI:** 10.3389/fbioe.2019.00206

**Published:** 2019-09-06

**Authors:** Christiane Laranjo Salgado, Beatriz Isabel Brites Teixeira, Fernando Jorge Mendes Monteiro

**Affiliations:** ^1^i3S–Instituto de Investigação e Inovação em Saúde, Universidade do Porto, Porto, Portugal; ^2^INEB–Instituto Nacional de Engenharia Biomédica, Universidade do Porto, Porto, Portugal; ^3^Faculdade de Engenharia (FEUP), DEMM, Universidade do Porto, Porto, Portugal; ^4^Institute of Health Sciences (ICS), Universidade Católica Portuguesa, Viseu, Portugal

**Keywords:** biomaterials, cryogel scaffold, collagen, nanohydroxyapatite, phosphoserine modification, guided bone tissue regeneration

## Abstract

In guided bone tissue engineering, successful ingrowth of MSCs depends primarily on the nature of the scaffold. It is well-known that only seconds after implantation, biomaterials are coated by a layer of adsorbed proteins/peptides which modulates the subsequent cell/scaffold interactions, especially at early times after implantation. In this work, nanohydroxyapatite and collagen based composite materials (Coll/nanoHA) were modified with phosphorylated amino acid (O-phospho-L-serine–OPS) to mimic bone tissue, and induce cell differentiation. The choice for this phosphorylated amino acid is due to the fact that osteopontin is a serine-rich glycol-phosphoprotein and has been associated to the early stages of bone formation, and regeneration. Several concentrations of OPS were added to the Coll/nanoHA scaffold and physico-chemical, mechanical, and *in vitro* cell behavior were evaluated. Afterwards, the composite scaffold with stronger mechanical and best cellular behavior was tested *in vivo*, with or without previous *in vitro* culture of human MSC's (bone tissue engineering). The OPS signaling of the biocomposite scaffolds showed similar cellular adhesion and proliferation, but higher ALP enzyme activity (HBMSC). *In vivo* bone ectopic formation studies allowed for a thorough evaluation of the materials for MSC's osteogenic differentiation. The OPS-scaffolds results showed that the material could modulated mesenchymal cells behavior in favor of osteogenic differentiation into late osteoblasts that gave raised to their ECM with human bone proteins (osteopontin) and calcium deposits. Finally, OPS-modified scaffolds enhanced cell survival, engraftment, migration, and spatial distribution within the 3D matrix that could be used as a cell-loaded scaffold for tissue engineering applications and accelerate bone regeneration processes.

**Graphical Abstract:**
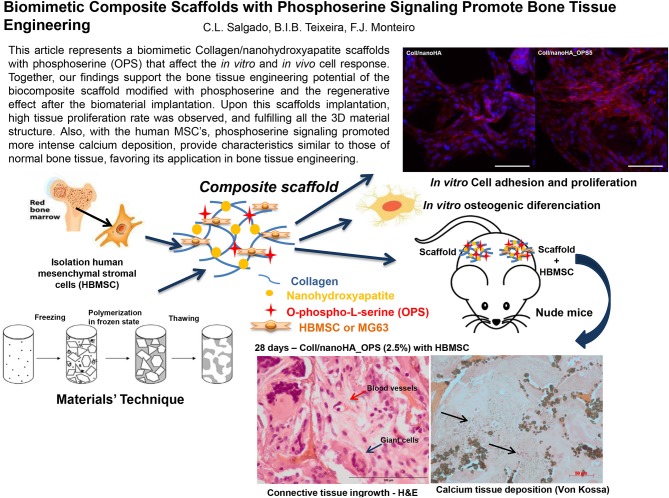
This article represents a biomimetic Collagen/nanohydroxyapatite scaffolds with phosphoserine (OPS) that affect the *in vitro* and *in vivo* cell response. Together, our findings support the bone tissue engineering potential of the biocomposite scaffold modified with phosphoserine and the regenerative effect after the biomaterial implantation. Upon this scaffolds implantation, high tissue proliferation rate was observed, and fulfilling all the 3D material structure. Also, with the human MSC's, phosphoserine signaling promoted more intense calcium deposition, provide characteristics similar to those of normal bone tissue, favoring its application in bone tissue engineering.

## Introduction

Bone is a mineralized connective tissue constituted by cells and a mineralized extracellular matrix (ECM) within which the majority of cells are contained (Jayakumar and Di Silvio, 2010). Mineralized ECM is composed of around 60% mineral, 30% organic matrix, 7% water, and <3% lipids (Clarke, 2008; Jayakumar and Di Silvio, 2010). The mineral content of bone is mostly hydroxyapatite, and the organic portion is mainly made of collagen, non-collagenous proteins, and proteoglycans (Clarke, 2008). Major bone defects are associated to trauma, osteonecrosis, tumor removal or congenital disorders, and the *gold standard* surgical treatment for reconstructing bone defects remains the use of bone autografts, but they are associated to high morbidity due to the need of obtaining considerable amounts of bone from donor-site and the area would be left for self-healing (Fernandez de Grado et al., 2018). However, commercially available products are unable to reproduce the complex bone structure (i.e., do not mimic the physical and biological properties of bone tissue), resulting in undesirable effects such as foreign body response, lack of regenerative capacity, degradation, and failure in long-term applications (Wang and Yeung, 2017). When designing novel regenerative solutions for bone regeneration application, it is essential to take into consideration defect-specific factors such as adequate mechanical strength, light weight and flexibility, a highly porous structure of a scaffold structure (Prasadh and Wong, 2018). The high swelling ratio and porosity, adequate mechanical, and biological properties of the scaffolds was supported by the association of bovine type I collagen (Coll) to synthetic nanoparticles of hydroxyapatite (nanoHA) (Rodrigues et al., 2013; Coelho et al., 2015; Salgado et al., 2016). This composite material has shown increased results in bone remodeling and a trend toward increased bone implant contact in the early ingrowth period (Zambonin and Grano, 1995; Wahl and Czernuszka, 2006; Rodrigues et al., 2013; Coelho et al., 2015). The scaffold should also afford sufficient void-space and self-organizing capabilities for cells to expand and promote rapid host vessel-infiltration upon implantation (Lovett et al., 2009). To guarantee adequate nutrients and oxygen supply throughout the scaffold, the cryogel technique (macropore forming technique), to produce the used scaffolds, should provide a porous structure with adequate pores size, and interconnectivity (Henderson et al., 2013).

The association of novel biomaterials and cell-therapies in tissue engineering strategies could offer new solutions for bone regeneration. Mesenchymal stem cells (MSC) hold great therapeutic potential due to their multi-lineage differentiation capacity, distinctive immunosuppressive properties and high expansion potential (Ullah et al., 2015). Transplanted MSC home to damaged tissues and inflammation sites, constituting a particularly promising population for regenerative medicine applications (Law and Chaudhuri, 2013; Wang et al., 2018). However, in living human tissues, stem cells or progenitor cells differentiation shall be induced according to biological clues that in natural tissues may be presented from proteins or other molecules naturally produced; when dealing with bone substitutes this molecular signaling must be introduced in the system (Chen and Liu, 2016). Non-collagenous proteins normally contain phosphorylated amino acid residues (e.g., osteopontin, bone sialoprotein, osteonectin). It is assumed that these residues are involved in binding protein to hydroxyapatite (Boskey, 1998). O-Phospho-L-serine (OPS) might be able to mimic the activities of a non-collagen proteins such as osteopontin (Hunter et al., 1996). It has been shown that OPS amino acids have a favorable influence by enhancing the *in vitro* viability of osteoblasts in these modified materials (Reinstorf et al., 2004; Raucci et al., 2014).

In this work, composite scaffolds were developed, based on collagen/nanohydroxyapatite, and phosphoserine with several concentrations. The OPS signaling into the scaffolds should affect the *in vitro* and *in vivo* response in terms of human bone cells adhesion and mesenchymal stem cells osteogenic differentiation. The overall goal of this work was to develop 3D scaffolds with enhanced regenerative potential and exceptional integration with adjoining tissue providing a superior treatment solution for bone regenerative clinical applications.

## Materials and Methods

### Preparation of Collagen-nanohydroxyapatite (Coll/nanoHA) Cryogels Modified With Phosphoserine (OPS)

Biocomposite cryogels (Coll/nanoHA−50/50% w/v) were produced similarly to previously work (Rodrigues et al., 2013; Salgado et al., 2016). Briefly, bovine collagen type I (Sigma-Aldrich, Germany) was homogenized (Ultra Turrax T25, IKA) at 10,000 rpm in 5 mM HCl at a concentration of 2% (w/v). The solution was mixed with a similar volume of 2% nanohydroxiapatite solution (nanoXIM.HAp202, FLUIDINOVA, S.A). Different ratios percentages of O-phospho-L-serine (OPS–(Sigma-Aldrich, Germany) were added to final solution (2.5 and 0.5%–OPS5 and OPS1 of total mass, respectively). All samples were crosslinked with 20 mM of N-hydroxysuccinimide (NHS–Sigma-Aldrich, Germany), and 40 mM of 1-Ethyl-3-(3-dimethylaminopropyl)carbodiimide (EDC–Sigma-Aldrich, Germany), and were kept in a freezer at −18°C for 24 h to complete the crosslinking. Subsequently, the scaffolds were washed with distilled water and finally dried in a freeze-dryer (Labconco, FreeZone 6) at −80°C for 24 h.

### Characterization of Cryogels

#### Morphological Studies

Morphology of cryogel samples was evaluated using scanning electron microscopy (SEM, FEI Quanta 400FEG) under secondary electrons mode. Collagen/nanoHA scaffolds with and without OPS were attached with Araldite^TM^ to an aluminum sample holder. Samples were then sputter-coated with palladium-gold alloy (Bal–Tec—SCD 050). Image analysis through ImageJ software (Wayne Rasband) was used to determine the scaffolds porous size range. The pore diameter for each sample was determined as:

$$\begin{array}{l} D={\left(\frac{A}{\pi }\;\right)}^{\frac{1}{2}} \end{array}$$

Where D and A were respectively, the pore diameter and the area of the circular projection of the pore in the image. The total number of pores analyzed for each material was over 200.

Mercury intrusion porosimetry method (Quantachrome Poremaster model No. 60) was used to evaluate total surface area, apparent density, and theoretical total porosity of all materials. The referred equipment allowed the detection of open pores in the range of 0.004–15.04 μm. Each scaffold was entered by mercury at high pressure, and data were obtained using Quantachrome Poremaster software.

#### Physico-Chemical Analysis

Dried samples (2 mg) were mixed with 200 mg of potassium bromide (KBr) and compressed into disks. Each disc was scanned at a resolution of 1 cm^−1^ over a frequency region of 400 to 4,000 cm^−1^ using a Fourier Transform infrared spectrophotometer (Perkin Elmer, USA) and the characteristic peaks of IR transmission spectra were recorded (average of 100 scans).

#### Mechanical Analysis

Dynamic Mechanical Analysis (DMA Tritec 2000, Triton Technology, UK) was carried-out in wet state (immersion in phosphate-buffered solution for 1 h), under dynamic compression solicitation. The scaffolds were subjected to increasing frequencies ranging from 0.1 to 15 Hz at room temperature. Four samples were tested for each type of scaffold to obtain the mean values.

### *In vitro* Biological Studies: Cellular Morphology, Viability, Proliferation and Differentiation

#### Cell Culture and Viability Assay

Coll/nanoHA (50/50% w/v) with and without OPS (2.5%) scaffolds (5 × 4 mm) were sterilized by ethylene oxide gas. Human bone osteosarcoma cell line (MG63, ATCC) was cultured in α-MEM (alpha modification of Eagle minimum essential medium, Sigma-Aldrich) supplemented with 10% fetal bovine serum (FBS, Gibco), 1% penicillin-streptomycin (3 × 10^−4^ mol/L and 5 × 10^−4^ mol/L, Gibco) and maintained at 37°C and 5% of carbon dioxide (CO_2_). After cell confluence, cells were seeded by drop method to Coll/nanoHA scaffolds with and without OPS 2.5% (1.5 × 10^5^ cells/scaffold). The evaluated time points were 7, 14, and 21 days for cellular viability and proliferation evaluation. Cell vitality was assessed with the non-toxic Alamar Blue dye [Resazurin, 10% (v/v)] assay. The supernatant was transferred to a black 96-well plate and fluorescence intensity was measured using a plate reader (Synergy, BioTek) at 530 and 590 nm for excitation and emission, respectively. The cellular metabolic activity results were normalized by the scaffold total DNA content (ng/mL) measured by the protocol showed at subsection Cellular proliferation assay. To observe cell differentiation, human bone marrow stromal cells (HBMSCs, Hospital São João, Portugal) that were isolated and maintained as described before (Salgado et al., 2014), and MG63 were cultured for 7, 14, and 21 days, without osteogenic supplements. Only on the control (2D culture–TCPS), osteogenic supplement (Control DEX) was added to the medium (0.1 mM dexamethasone, 0.1 mg.mL^−1^ ascorbic acid, and 10 mM b-glycerophosphate).

#### Cellular Morphology

For morphology analysis, the samples were fixed with 4% paraformaldehyde (Sigma) for 30 min and then washed in PBS (Sigma-Aldrich). Then, the materials were incubated for 10 min with 0.1% Triton X100 solution (Sigma-Aldrich), washed with 1% bovine serum albumin solution (BSA, Sigma-Aldrich), and the cytoplasm were stained with Alexa Fluor-conjugated Phalloidin 594 nm (Invitrogen) at 2.5% for 1 h at room temperature and protected from the light. Nuclei were stained with DAPI (4′-6-diamidine-2-phenylindole at 0.2%, Invitrogen) for 5 min. For focal adhesion immunostaining, same protocol were performed for the cell membrane's permeabilization and to block non-specific binding. Samples were then incubated with mouse anti-human vinculin mAb clone hVIN-1 (Sigma) at a 1:100 dilution for 1 h at room temperature and stained with Alexa Fluor 594 rabbit anti-mouse IgG, F(ab′)2 fragment (Molecular Probes) at a 1:400 dilution for 1 h at room temperature. Samples were subsequently washed and nuclei were counterstained with 1 μg mL^−1^ DAPI (Molecular Probes) for 10 min at room temperature. Immunostained cells were analyzed in three replicates of each scaffold. To evaluate non-collagenous bone protein, HBMSC cells were culture for 21 days without osteogenic supplements, and the secreted human osteopontin (OPN) were stained with rabbit anti-human osteopontin (AB 1870, Merck, 1:500), followed by Alexa Fluor 488 goat anti-rabbit IgG secondary antibody (Invitrogen Molecular Probes, 1:1,000) for 1 h each antibody, at room temperature. To conclude, the images were acquired with a Leica SP2 AOBS SE camera, with the excitation lasers of 405, 488, and 594 nm.

#### Cellular Proliferation Assay

DNA content was measured using the Quant-iT^TM^ Picogreen® DNA assay (Invitogen, UK) according to the company's instructions. After the time-points, scaffolds were incubated with 0.5 ml of ultra-pure water at 37°C for 1 h. Subsequently, they were put in a freezer at −80°C for 1 h and then thawed at room temperature. The supernatant with the lysed cells were collected and incubaded with Picogreen® solution for 1 h. Afterwards, the fluorescence intensity was measured with a microplate spectrofluorometer (SynergyMx, BioTek) at 480 and 520 nm excitation and emission, respectively.

#### Cellular Differentiation Assay

Once the time points were reached, lysed cells supernatant of the samples were prepared as described above. The ALP enzyme activity was followed through substrate hydrolysis, using p-nitrophenol phosphate (Sigma-Aldrich) at pH 10. After incubation, the reaction was stopped by adding NaOH (1M, Sigma) and p-nitrophenol was quantified at 405 nm, using a plate reader (BioTek).

#### Osteogenic Phenotype Analyses Through mRNA Expression of Runx-2, Osteopontin (OPN), BMP-2 and Osteocalcin (OC)

Total RNA was extracted from HBMSC with NucleoSpin kit (Macherey-Nagel), as recommended by the manufacturer. Subsequently, cDNA synthesis was obtained with the iScript™ cDNA Synthesis Kit (BioRad) as recommended by the manufacturer. After cDNA synthesis reaction, quantitative real-Time PCR was carried out in mixture containing 1 μL of cDNA, 10 μM of each forward and reverse primers (Runx-2, BMP-2, Osteopontin–OPN, and Osteocalcin–OC, Table 1) and 10 μL of iTaq™ Universal SYBR® Green Supermix (BioRad). qPCR experiments were run using an iQ5 (BioRad) and analyzed with the iCycler IQ software. The housekeeping gene glyceraldehyde 3-phosphate dehydrogenase (GAPDH) was used as the endogenous assay control. Relative quantification of gene amplification by qPCR was performed using the cycle threshold (Ct) values and relative expression levels were calculated using the 2^(-ΔΔ*CT*)^ method. The expression value for each target gene (Rux-2, BMP-2, OPN, and OC) was normalized to the GAPDH value at each time point. Results were normalized to the Coll/nanoHA average results (for day 7 and 14), and are represented as fold change. Positive control with osteogenic supplementation (Control—DEX) were also evaluated. For each PCR, samples were analyzed in duplicate and three independent experiments were performed.

**Table 1:** Primers for PCR amplification.

**Gene**	**Primer sequence (forward)**	**Primer sequence (reverse)**
GAPDH	5′-TAACTGGTAAAGTGGATATTG-3′	5′-GAAGATGGTAGATGGATTTC-3′
Runx-2	5′-GTGCCTAGGCGCATTTCA-3′	5′-GCTCTTCTTACTGAGATGGAAGG-3′
OC	5′-AGAGTCCAGCAAAGGTGCAG-3′	5′-TCAGCCAACTCGTCACAGTC-3′
BMP-2	5′-GACGAGGTCCTGAGCGAGTT-3′	5′-GCAATGGCCTTATCTGTGAC-3′
OPN	5′-ACTCGAACGACTCTGATGATGT-3′	5′-GTCAGGTCTGCGAAACTTCTTA-3′

#### *In vivo* Biological Studies: Histology and Immunohistochemical Analysis

##### Animal model protocol

HBMSC cells were seeded into Coll/nanoHA scaffolds with and without phosphoserine (OPS5−2.5%) (5 mm diameter and 4 mm of high) and cultured in α-MEM osteogenic complete medium (0.1 mM dexamethasone, 0.1 mg.mL^−1^ ascorbic acid, and 10 mM b-glycerophosphate) for 7 days. Afterwards, blank scaffolds (with and without OPS5), and scaffolds seeded with cells (1.5 × 105 HBMSCs per scaffold) were subcutaneously transplanted into the dorsae of four nude female mice, 6 week-old (i3S animal house, Portugal). The study was performed in accordance with Animal based studies Ethical Committee and fulfilled all legal requirements (Approved by the Ethical Animal Commission of i3S, Portugal). Surgical procedures were performed under standard aseptic conditions. Animals were anesthetized with 3–5% isoflurane for induction and 1–2% for surgical procedures. A midline incision through the dorsal skin was performed and two subcutaneous pockets were created, one on the left side (scaffolds with HBMSCs) and one on the right (scaffolds without cells) side. The dorsal wound was then closed with surgical staples. After recovery, the mice were caged in pairs and allowed to move in their cages without restriction. They were fed with commercial mice chow and water for 1, 2, and 4 weeks ad lib. After the foreseen period of time, the mice were euthanized with carbon dioxide asphyxiation.

##### Histology analysis

All samples were explanted and fixed in 10% neutralized buffered formalin for 24 h and then processed for histology. Fixed samples were embedded in paraffin and were sectioned longitudinally with a microtome (5 mm of thickness). The sections were stained with hematoxylin-eosin (H&E), Alizarin red and Von Kossa (sites of tissue calcium deposition) for light microscopy examination. Image analysis through ImageJ software (Wayne Rasband) was used to determine the total tissue ingrowth area (H&E) and percentage of mineralized tissue (Von Kossa). For the evaluation of such parameter, over 50 images of each explant were used (4 replica).

##### Immunohistochemistry analysis

Immunohistochemistry analysis were performed to follow the HBMSC presence into the scaffold and secretion of human osteopontin (OPN) after 4 weeks of implantation. For that assay, human nuclei, and OPN were probed after antigen recovery. With this purpose, masked epitopes were exposed by treatment with citrate buffer (pH 9) for 20 min at 97°C. Sections were incubated with mouse anti-human nuclei primary antibody (MAB4383-3 E1.3 Millipore, 1:400, overnight at 4°C) and rabbit anti-human osteopontin (AB 1870, Merck, 1:500). This procedure was followed by 1 h incubation with Alexa Fluor 594 goat anti-mouse IgG secondary antibody (Invitrogen Molecular Probes, 1:1,000) and Alexa Fluor 488 goat anti-rabbit IgG secondary antibody (Invitrogen Molecular Probes, 1:1,000). All slides were mounted in Vectashield™ with DAPI (Sigma). Images were obtained using a fluorescence inverted microscope (Axio Imager Z1, Zeiss). Image analysis through ImageJ software (Wayne Rasband) was used to determine the percentage of human cells into the implanted scaffolds (with or not OPS) after 4 weeks (anti-human nuclei antibody). For the evaluation of such parameter, over 20 images of each explant were used (4 replica).

## Statistical Analysis

Data were presented as mean ± standard deviation (*n* = 3) and they were analyzed using the one way ANOVA test. Differences between groups were considered statistically significant for *p* < 0.05.

## Results

### Characterization of Cryogels: Morphological, Physico-Chemical and Mechanical Studies

To test the hypothesis that Collagen/nanohydroxyapatite with O-phospho-L-serine (OPS) can induce cellular differentiation and bone regeneration, Coll/nanoHA_OPS scaffolds were produced and characterized *in vitro* for physico-chemical and mechanical properties. Also, the material's biocompatibility and cellular modulation on *in vitro* cell culture assays and in a mouse ectopic bone formation model were tested.

**Figure 1:**
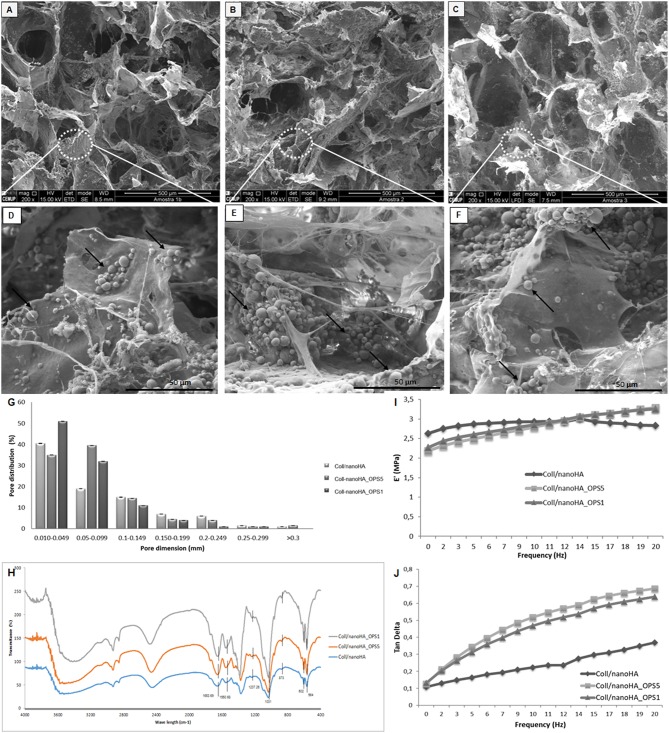
Scanning electron micrographs (SEM) of **(A,D)** Coll/nanoHA scaffold, **(B,E)** Coll/nanoHA_OPS5 (OPS−2.5%) scaffold, **(C,F)** Coll/nanoHA_OPS1 (OPS−0.5%) scaffold. Scale bar: 500 μm **(A–C)** and 50 μm **(D–F)**. **(G)** Pore distribution for Coll/nanoHA biocomposite scaffolds with and without OPS. **(H)** FTIR spectra of Coll/nanoHA scaffold, Coll/nanoHA_OPS5 scaffold, and Coll/nanoHA_OPS1 scaffold. Storage modulus **(I)** and Tan delta **(J)** under dynamic compression solicitation vs. increasing frequency, ranging from 0.1 to 10 Hz. Control–Coll/nanoHA scaffolds.

In this work, Coll/nanoHA_OPS scaffolds were produced using cryogel methodologies, as previously described for other materials (Lozinsky et al., 2003; Kathuria et al., 2009). To determine their structure morphology and pores distribution, all scaffolds were observed using scanning electron microscopy (SEM). Figures 1A–F shows the SEM images of Coll/nanoHA scaffolds with and without phosphoserine present in different concentrations (2.5%–OPS5 and 0.5%–OPS1).

The nanoHA aggregates were homogeneously dispersed throughout the scaffold pore surface (Figures 1D–F—black arrows). Overall, all biocomposite scaffolds presented heterogeneous porous network with macroporosity ranging from 150 to 400 μm and micropores between 10 and 100 μm in diameter. Thus, pores between 10 and 405 μm were measured by the software, with an average pore size of 89.97 ± 75 μm for Coll/nanoHA scaffold, 23.95 ± 65 μm for Coll/nanoHA_OPS5 scaffold, and 18.2 ± 60 μm for collagen-nanoHA_OPS1 scaffold. The presence of higher concentration of OPS reduced the theoretical total porosity (75.77% for Coll/nanoHA and 52.69% for Coll/nanoHA_OPS5—Table 2), but enhanced the total surface area (64.05 m^2^/g for Coll/nanoHA and 70.79 m^2^/g for Coll/nanoHA_OPS5—Table 2). Pore size distribution for all the scaffolds is shown in Figure 1G.

**Table 2:** Pore size, surface area, and porosity characterization for Coll/nanoHA biocomposite scaffolds with and without OPS.

**Materials**	**Coll/nanoHA**	**Coll/nanoHA_OPS5**	**Coll/nanoHA_OPS1**
Maximum pore diameter (μm)	391	459	346
Minimum pore diameter (μm)	15	2	18
Average pore diameter (μm)	89.97 ± 75	23.95 ± 65	18.2 ± 60
Surface Area (m^2^/g)	64.05	70.79	23.09
Theoretical porosity (%)	75.77	52.69	45.93
Apparent density (g/cc)	0.54	0.32	0.15

The spectrum of collagen chains (Figure 1H) exhibited typical proteins bands: amide I (C = O stretching) at 1,658 cm^−1^, amide II (N-H deformation) at 1,550 cm^−1^, and amide III (N-H deformation) at 1,237 cm^−1^ (Chang and Tanaka, 2002). The hydroxyapatite characteristic peaks were observed by deconvolution: ν1 P—O symmetric stretch at 873 cm^−1^; ν3 P—O asymmetric stretch, triple degenerate at 1,031 cm^−1^; ν4 O—P—O bend, triple degenerate at 564 cm^−1^. Amide I band on the Coll/nanoHA with and without OPS appeared at 1,663 cm^−1^. This band represented the stretching vibration of C = O and the modification of the band could be probably an indication of a link of Ca^2+^ from nanoHA with collagen. This result could infer that some nanoHA aggregates had interactions with the collagen surface through the carbonyl groups present on the natural structure of the polymer (Wenpo et al., 2015). No difference between the above referred peaks in the scaffolds with different concentrations of OPS and the materials without the modification was observed.

The mechanical properties of the composite scaffolds were evaluate by DMA. For these cryogels, based on collagen and nanohydroxyapatite, the storage modulus (E′) is about one order of magnitude higher than the loss modulus (E″) indicating an elastic nature of the biocomposite scaffolds (Rodrigues et al., 2013). The storage modulus and tan delta behavior vs. the loading cycle frequency is reported in Figure 1I. As it may be seen in the image, at low frequencies, E′ values decreased in the presence of low and high amounts of OPS when compared to the control (without phosphoserine). But, at higher frequencies (>10 Hz), the presence of phosphoserine increased the materials' stiffness. The loss factor equation tan δ = E″/E′, indicates the ability of dispersing the cyclic mechanical energy as heat. The respective results were plotted in Figure 1J.

### *In vitro* Biological Studies: Cellular Morphology, Viability, Proliferation and Differentiation

**Figure 2:**
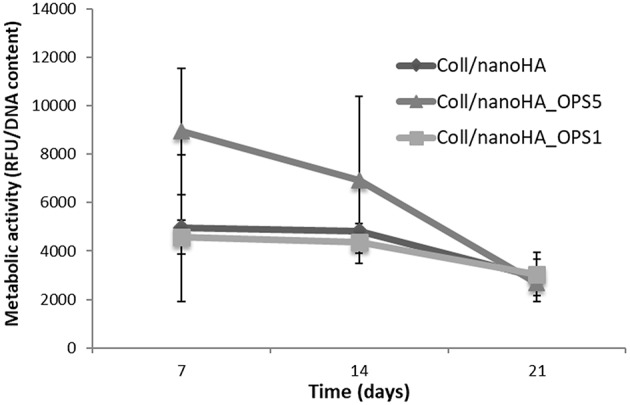
HBMSC cellular viability after cultured on Coll/nanoHA (50:50 wt%) with or without OPS (2.5 and 0.5%) for 7, 14, and 21 days. The cellular metabolic activity results were normalized by total DNA content (ng/mL) quantified in each time point.

**Figure 3:**
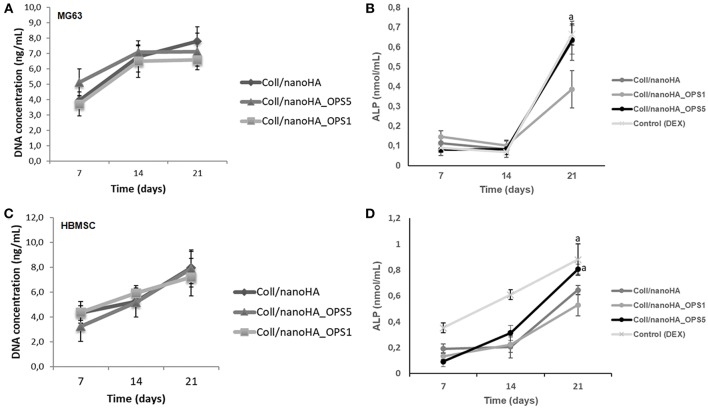
DNA concentration and cells ALP activity after cultured on Coll/nanoHA (50:50 wt%) with or without OPS5 for 7, 14, and 21 days (**A**, **B**–MG63 cells; **C,D**–HBMSCs). Statistically significant **(A)** *p* < 0.05.

HBMSC's showed a tendency of higher cellular metabolic activity into the scaffolds with higher concentration of OPS (2.5%) after 7 and 14 days of culture (Figure 2). After 21 days, the cellular metabolic activity decreased to similar values of the scaffolds with lower OPS concentration and without the modification (Coll/nanoHA. Although, human osteoblasts (MG63) and HBMSC's seeded onto the different cryogels showed increasing profile in cell proliferation with the time points of *in vitro* culture (Figures 3A,C, respectively), following a similar time-dependent profile. With the MG63 cell line culture, the effect of the phosphoserine signaling enhanced the proliferation when compared with the scaffold without OPS, as shown by DNA concentration after 14 days (Figure 3A). For the HBMSC cell culture, after 7, and 14 days, there were no statistical differences between the scaffolds, even with the presence of lower and higher concentration of phosphoserine (Figure 3C).

In cellular differentiation analysis, increased in ALP enzyme activity is associated to an early osteoblastic phenotype. Even without the addition of osteoinduction supplements (i.e., dexamethasone), after 3 weeks, human bone cells (MG63) in the scaffolds with higher concentration of phosphoserine (OPS5) expressed ALP activity values similar to those of the positive control (2D cell culture with osteogenic supplementation–dexamethasone) as showed in Figure 3B (Control DEX). Also, in basal conditions, only at day 21, ALP activity was significantly higher for HBMSCs cultured into Coll/nanoHA_OPS5 scaffolds. Similar values were also observed with the osteogenic induction control (dexamethasone). But, lower ALP values were observed with both cell cultures (MG63 and HBMSC) after 21 days into Coll/nanoHA_OPS1 scaffolds when compared to simple scaffolds of Coll/nanoHA (Figure 3D). The scaffolds with lower OPS percentage showed similar cellular viability, but slightly lower cellular proliferation and differentiation induction (ALP activity) after 21 days. These findings seem to indicate that only higher concentration of OPS (2.5%) immobilized in the scaffold porous surface was able to stimulate osteogenic differentiation, so only this modified scaffold was evaluated by qPCR and implanted *in vivo* (subcutaneous animal model).

**Figure 4:**
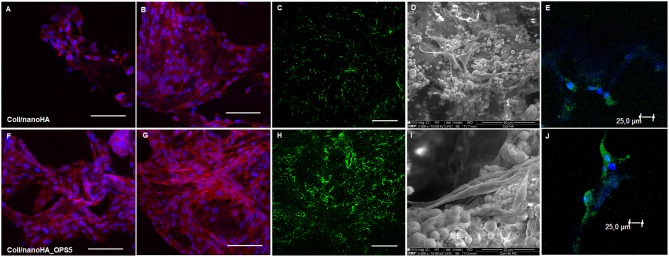
Confocal microscopy images of HBMSC cells cultured for 7 and 21 days on Coll/nanoHA **(A,B)** and Coll/nanoHA_OPS5 **(F,G)** scaffolds (blue: nuclei, red: cytoplasm). Human osteopontin immunostaining (green) for HBMSC cell culture after 21 day **(C)** Coll/nanoHA and **(H)** Coll/nanoHA_OPS5. Scale: 100 μm. SEM images of 7 days in culture with Coll/nanoHA and Coll/nanoHA_OPS5 **(D,I)**. Immunostaining of focal adhesions (green and blue–DAPI) of HBMSC cells cultured for 1 day on Coll/nanoHA **(E)** and Coll/nanoHA_OPS5 **(J)**.

Confocal microscopy images of human bone marrow stromal cells (HBMSC) seeded on Coll/nanoHA biocomposite scaffolds with and without phosphoserine (OPS5) were obtained to observe cells biocompatibility behavior (Figure 4). After 7 days of culture, it was observed that the cells presenting star shaped morphology were adherent and well-attached on all the different samples and for the different time points. Moreover, it was observed that after 7 days, the highest cell viability and density were found for the biocomposite cryogels with phosphoserine, where the cells were well-flattened, showing many cell membrane extensions (Figures 4A,B,F,G), cell-to-cell and cell-material surface contact points (Figures 4I,J). At day 21, both biocomposite scaffolds surfaces and macropores access walls were almost completely covered by cells that formed continuous cell layers in some regions. Besides, it should be observed that cell ingrowth seemed to follow the irregularities of the materials' surface, covering both scaffolds pore walls (Figures 4B,G). These cells also secreted ECM with bone proteins (osteopontin) after 21 days, showing their capacity to differentiate *in vitro* within the scaffolds (Figures 4C,H). The HBMSC's cultured into the scaffolds with OPS signaling showed higher presence of human OPN on the material's surface (Figure 4H).

**Figure 5:**
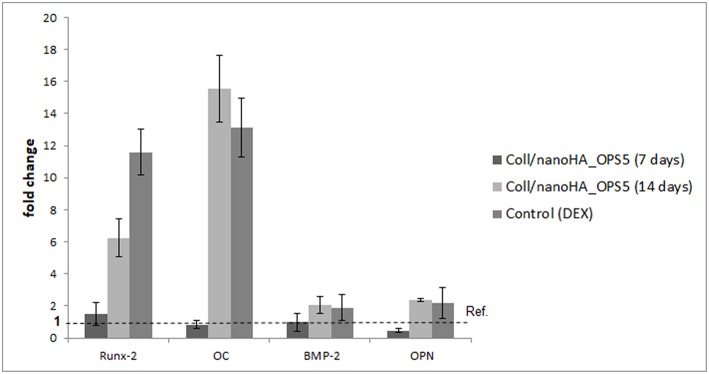
qPCR of HBMSC expression after cultured on Coll/nanoHA (50:50 wt%) with OPS5 for 7 and 14 days. It was used the ΔΔCt method with GAPDH gene expression as an endogenous reference.

To evaluate the osteoinductive capacity of these materials, human mesenchymal stromal cells were culture into the scaffolds with or without phosphoserine in the absence of dexamethasone (osteogenic inductor). The HBMSC cells gene expression observed on quantitative real-time PCR after 7 days showed higher levels of osteogenic marks (Runx-2, OPN, BMP-2, and osteocalcin—Figure 5) in the presence of OPS. Therefore, after 14 days, high gene expression of bone tissue differentiation (OC, OPN, and Runx-2) could be observed only for the cells cultured on Coll/nanoHA_OPS5 and the positive control with osteoinduction supplements (dexamethasone–DEX).

### *In vivo* Biological Studies: Histology Morphometry and Immunohistochemical Analysis

**Figure 6:**
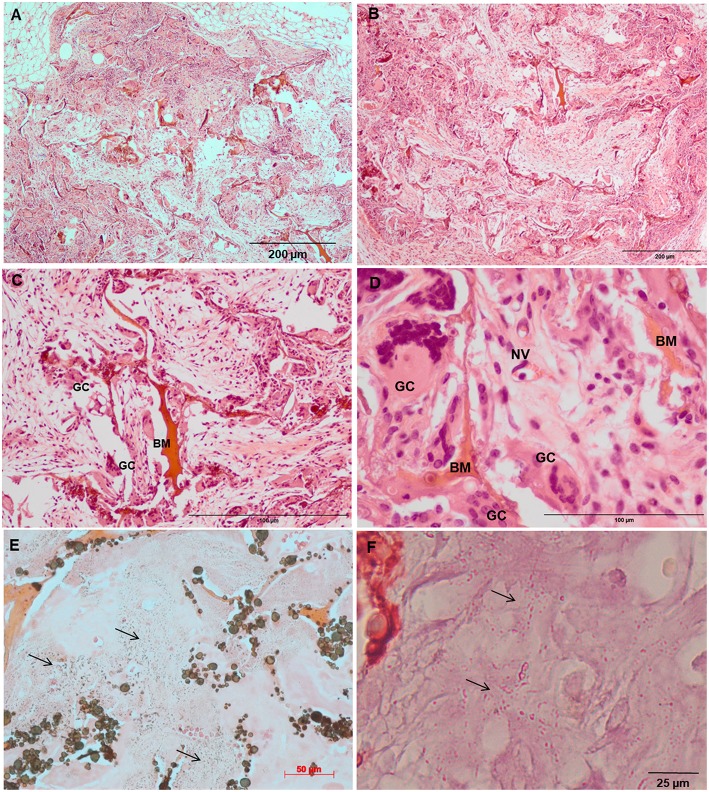
Tissue ingrowth within a Coll/nanoHA after 14 days **(A)**, scaffold with HBMSC culture implanted after **(B)** 14 days (100 μm), **(C,D)** 28 days (50 μm). **(E,F)** Von Kossa and Alizarin red. Black arrows—Calcium crystals. BM—Biomaterial; GC—Giant cells; NV—new blood vessels.

**Figure 7:**
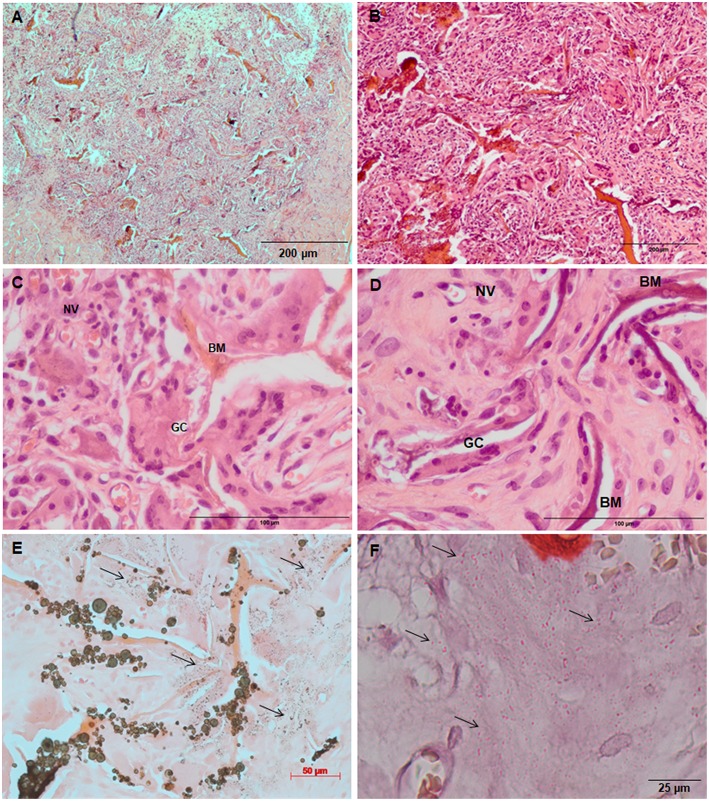
Tissue ingrowth within a Coll/nanoHA_OPS5 after 14 days **(A)**, scaffold with HBMSC culture implanted after **(B)** 14 days (100 μm), **(C,D)** 28 days (50 μm). **(E,F)** Von Kossa and Alizarin red. Black arrows—Calcium crystals. BM—Biomaterial; GC—Giant cells; NV—new blood vessels.

**Figure 8:**
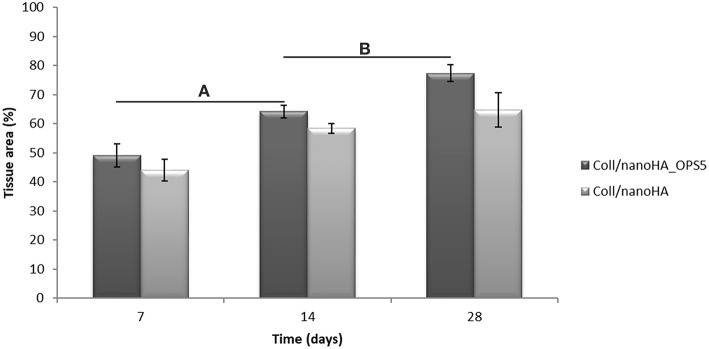
3D Coll/nanoHA scaffolds with and without OPS implants after 7, 14, and 28 days (percentage of the total section area—μm^2^). Statistically significant **(A,B)** *p* < 0.05.

Increased tissue ingrowth was observed for the subcutaneous implants without cell-loaded *in vitro* culture for modified biocomposite materials (Coll/nanoHA_OPS5) similar to the *in vitro* analysis (Figures 6A, 7A). Scaffolds with HBMSC's culture showed higher connective tissue ingrowth when compared to the empty implanted materials (Figures 6B–D, 7B–D). The total connective tissue ingrowth area was calculated within the artificial 3D scaffolds implanted after 7, 14, and 28 days (Figure 8). The results show that the materials modified with phosphoserine induced the continuous growth of the surrounding tissue inside the porous scaffold, while without the OPS, the total tissue area was not statistically different between 2 and 4 weeks after surgery (Figure 8). In the same *in vivo* subcutaneous model, it was possible to observe the presence of giant cells and inflammatory cells such as macrophages surrounding the apatite particles. Comparable results were also observed by Baskin et al. (2012).

Similarly, the materials with and without OPS signaling, after 28 days, showed a visible calcium deposition on the subcutaneous matrix only on the implants loaded with HBMSC's (Figures 6E,F, 7E,F). Nanohydroxyapatite particles and tissue calcium deposition could be observed with Von Kossa staining and Alizarin red (dark brown and red dots–Figures 6, 7E,F, respectively). These calcium crystals staining images showed that more intense MSC's osteogenic differentiation occurred when OPS modified scaffolds were implanted subcutaneously without the addition of osteogenic synthetic factor such as dexamethasone (Yoshikawa et al., 1996). More mineralized tissue formation was evident in the periphery of the scaffold, which could be directly related to the *in vitro* seeded HBMSCs and the material's incubation for 7 days. These histological results were confirmed by the semi-quantitative analysis of the implant slices (Figure 8). The total area of these calcium precipitates and the relative percentage of area were calculated, and the scaffold with phosphoserine showed almost 5 times and 2 times higher, respectively, than the samples without the OPS signaling. Without the cells, there was no statistical difference between the different materials (Figure 8 and Table 3).

**Table 3:** After 28 days of scaffolds subcutaneous implantation, connective tissue calcification (calcium tissue deposition) reached the total area calculated by ImageJ software in μm^2^.

**Cells**	**Materials**	**Total area (μm^**2**^)**	**% of area**
–	Coll/nanoHA_OPS5	321.7 ± 34.5	0.34 ± 0.11
–	Coll/nanoHA	163.5 ± 62.6	0.18 ± 0.09
HBMSC	Coll/nanoHA_OPS5	556.1± 137.3	0.59 ± 0.15
HBMSC	Coll/nanoHA	108 ± 68.8	0.14 ± 0.042

**Figure 9:**
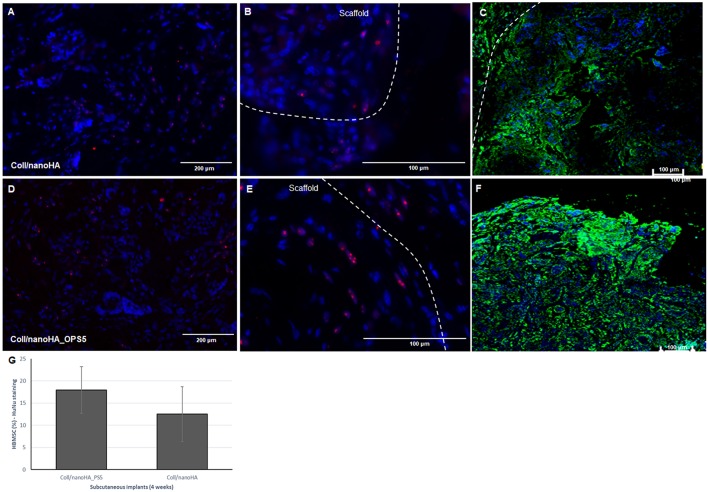
Connective tissue ingrowth within Coll/nanoHA **(A–C)** and with OPS5 **(D–F)** with HBMSC culture implanted for 28 days. Mouse cells nuclei in blue, HuNu in pink (human nuclear staining), and OPN in green. **(G)** Quantitative image analysis of human MSC's presence (percentage) into the scaffolds with and without OPS signaling after 4 weeks of implantation.

After immunostaining the human cells in the animal implants, it could be observed that in all the pre-cultured scaffolds implanted for 28 days, higher amount of human MSCs were still present inside the materials. Analyzing the tissue inside the scaffolds and surrounding it, it was possible to observe the human MSC's migrating toward the mouse tissue (Figures 9A,B,D,E). Thus, the number of HBMSC cells number inside the biocomposites with phosphoserine after 28 days were considerably higher into a mouse tissue (Figure 9G) but not statistically different when compared to the control material. The human proteins secreted by the HBMSC were evaluated by the detection of human ostepontin (OPN) inside the scaffold tissue. The presence of the protein is more evident on the periphery of the OPS- modified scaffolds, in accordance with the higher number of cells in this area (Figures 9C,F).

## Discussion

The preservation of adequate structural integrity of the scaffold is critical in tissue engineering and regenerative applications; besides, cell and tissue growth and remodeling are important to reach stable biomechanical conditions at the defect site. Furthermore, in the case of low rate tissue regeneration process such as bone, the material must provide enough transient mechanical support to withstand *in vivo* stresses and loading (Malafaya et al., 2008). Consequently, mechanical properties of the materials before implantation are important elements for their subsequent long-term success or failure (Kelly and Prendergast, 2006). HA nanoparticles powder, composed by aggregates of nanophased crystals with size around 100 nm and a surface area over 120 m^2^/g was obtained to enhance the mechanical properties of the collagen/nanoHA biocomposite scaffolds (Coelho et al., 2015). These particles aggregates morphology has been previously studied (Laranjeira et al., 2010) and a wide range of diameter sizes of spherical nanoHA aggregates were observed. Detailed information about the material composition could be found in a US patent (US 2017/0042936A1 Feb. 16, 2017). These particles adhesion to the collagen fibers was very important because particles detachment could cause significant problems, such as released particles could migrate to the surrounding tissues and induce a chronic inflammatory response in the host organs and tissues, or it could never be released or rapidly dissolved and would remain within the bone defect (Sena et al., 2009). In this work the composite scaffolds were produced by cryogelation method. As described for cryogelation (Lozinsky et al., 2003; Kathuria et al., 2009), the formation of water crystals (solvent) during freezing process led collagen and EDC/NHS to stay in a liquid phase forming stable chemical bonds (crosslink). After thawing, pores with heterogeneous size and geometry were presented in the natural polymer. These scaffold production technique also showed a highly interconnective porous structure (three-dimensionally). Highly interconnective structure was chosen to homogenize the cell distribution within the material, and also to enhance the nutrients and metabolites diffusion inside the scaffold. Overall, Coll/nanoHA scaffolds structure presented heterogenous porous network with macro-porosity typically above 200 μm in diameter, and interconnecting micropores (about 10 μm). It has been published that an ideal porous biomaterial should exhibit both micro and macro-porosity (Rücker et al., 2008; Singh et al., 2009; Wei et al., 2010). Pores size below 10 μm could be essential to transfer of oxygen, nutrients, and metabolites and also for cell-ECM interactions (cell adherence), and pores over 100 μm in diameter could favor *in vivo* tissue ingrowth into the scaffolds (Fierz et al., 2008). DMA analysis of Coll/nanoHA_OPS scaffolds were performed in wet state, because more accurate data of the mechanical properties could be gained from the natural polymer and also to better mimic the physiological conditions in which the materials should potentially be used. In the analysis, both storage and loss modulus (E′ and E″) were measured between 0.1 and 20 Hz of frequency, which are typical low and high solicitation described in physiological situations in load-bearing applications (Roach et al., 2007; Chen et al., 2012). Regarding the dynamic mechanical evaluation, composite scaffolds with phosphoserine showed extraordinary tension dissipation ability, related to hindering properties, especially at low and high frequencies, which can be a very useful feature in view of *in vivo* implantation (Zadpoor, 2017). Significant differences on the tan delta results were observed between the cryogels with and without OPS. Therefore, it could be concluded that the material became more viscous (permanent deformation) and less elastic in the presence of phosphoserine modification.

When the cells were into the biomaterials structure, they should have some morphological changes in order to stabilize the cell-surface interface. In the occurrence of cell adhesion to the materials' surface, the most important results relates to a more organized cellular network, cell membrane elongation with filopodia extensions (Khalili and Ahmad, 2015). Consequently, not only the chemical surface, but also topology is an important aspect that interferes on the cellular behavior (Beringer et al., 2015). After cellular contact with the scaffolds signaling OPS (MG63 and HBMSC), there were no differences in their morphology, adhesion capacity and proliferation, when compared to the control scaffold (Coll/nanoHA). Human bone marrow cells had a progressive differentiation pathway that start with pre-osteoblasts and later became osteoblasts. Once the final differentiation state was reached, cells could secrete ECM proteins for the matrix mineralization (Wang et al., 2016). Therefore, the HBMSC's revealed a tendency to a decrease in the cellular metabolic activity (Figure 2), but these results are similar to findings published in the literature. Some authors stated that with osteoinduction, the human MSC's entered in the stage of osteoblastic differentiation, showing lower level of cell proliferation and metabolic activity. The researchers presented that all the materials that exhibited decreasing levels of proliferation also showed increasing levels of differentiation, increased ALP activity and osteocalcin (OC) gene expression levels (Schwartz et al., 1999; Carvalho et al., 2016). Indeed, it was observed an increasing of cellular proliferation (Figure 3C), along the studied period, this behavior was a positive result that should end-up by leading to a normal tissue growth for the proposed clinical application.

The ability of human MSCs to produce different lineages is now a well-known concept and their osteogenic differentiation potential has become one of the most researched topics in bone regeneration (Abbah et al., 2008). Encouraging results have been obtained when using osteoblasts differentiated from MSCs in bone regenerative processes (Healy and Guldberg, 2007; Moioli et al., 2007). Along these findings and targeting at developing a bone tissue engineering approach, the differentiation of immobilized HBMSCs toward the osteogenic lineage was explored by analyzing several cell differentiation markers. First, the activity of ALP enzyme, an early osteoblastic marker “infer” the stage of cell differentiation. The activity of this enzyme increases during synthesis of the extracellular matrix, which matches to the beginning of cell differentiation (Stein et al., 1990). This *in vitro* test could disclose the osteoconductive effect for phosphoserine and its essential role on the mesenchymal stromal cells differentiation (Supplementary Material), which could be observed in the enhancement of the ALP enzyme activity on early times of cells cultures only for the OPS modified scaffolds. The osteoconductive effect were also observe in CaP cements modified with phosphoserine that clearly enhanced cell line differentiation showed by alkaline phosphatase activity and expression of typical osteogenic markers (Raucci et al., 2014).

Many transcription factors are responsible for the commitment of multipotent mesenchymal cells into the osteoblast cell lineage. A valuable factor is Runx-2, that is able to up-regulate the expression of the main bone ECM protein genes, such as osteopontin and osteocalcin, leading to a higher number of immature osteoblasts from multipotent stem cells. The advanced development of the osteoblastc phenotype from a non-differentiated cell to an established osteoblastic cell is considered by a sequence of specific genes expression that remain over three periods of osteoblast phenotype development: proliferation, in a sequence of maturation and ECM synthesis, and as a final point, the mineral deposition onto the matrix (Neve et al., 2011). Many works propose that osteocalcin (OC) is involved in the mineral deposition regulation and that it acts as a signal for osteoblast activation and differentiation (Chenu et al., 1994), confirming that OC is a biomarker for mature osteoblasts (Lian et al., 2001). Concerning the cells gene expression and osteogenic markers, the results indicated that the osteoblast gene markers could be readily regulated in an ECM-mediated way. Cell contact with ECM proteins is very significant on regulating the osteogenic differentiation of human MSCs (Sun et al., 2018). Our data strongly suggested through the real-time PCR results, that the presence of phosphoserine enhanced the expression of osteogenic gene markers, as early as 7 days in culture even without any osteogenic supplement (dexamethasone). Those results showed similar gene expression when compared to a positive control of a 2D cell culture with osteogenic induction culture medium (dexamethasone). These findings also corroborate to the *in vitro* results that showed bone proteins presence (OPN) in the HBMSC's ECM after 21 days. Therefore, the scaffolds' osteoconduction effect on the HBMSC's were higher observed with the presence of OPS molecules.

The mouse subcutaneous model is commonly accepted as the ideal initial model for investigating the *in vivo* behavior of bone substitutes (Yokoyama et al., 2005; Tommila et al., 2008). For this study, 1, 2, and 4 week duration period were selected because the intent was to assess the material performance during short-term inflammation (1 and 2 weeks), and also evaluate the response after 4 weeks, to observe when could occur a significant *in vivo* degradation of the material (Tommila et al., 2008). After implanted Coll/nanoHA_OPS scaffolds, the histological evaluation was performed at day 7 revealed that the scaffold structure was still present. The maintenance of the 3D structure should be related to the chemical bonding of the collagen fibrils allowed by the crosslinking step (EDC/NHS). After the *in vivo* implantation, we could observe porous scaffold architecture similar to a non-implanted material. Besides, the histological findings indicate that Coll/nanoHA supported tissue ingrowth and new micro-vascularization. Importantly, in the context of tissue healing, granulation tissue replaces hematoma during the early stages of tissue repair. Previous reports showed that Coll/nanoHA cell-binding domains could promote *in vivo* migration and proliferation (Rodrigues et al., 2013; Salgado et al., 2016) of animal fibroblasts, an important cell population involved in granulation tissue formation and wound healing (Micallef et al., 2012). It was observed that the presence of phosphoserine not only enhanced the percentage of the total tissue ingrowth along the time-points, being statistically different from the material without the OPS after 14 and 28 days (Figure 8). Four weeks after implantation, newly ECM with human OPN and calcium deposition was observed, only for materials cultured with the HBMSCs seeded and cultured *in vitro* for 7 days (Figures 6F, 7F, 9E). In some animals, the presence of residual highly vascularized soft tissue inside the implant could still be observed. The bone-like tissue formation was only observed in the periphery of the scaffold, which may be related with the fact that higher number of HBMSCs adhered and proliferated on the periphery after 7 days of *in vitro* culture. Also, calcium tissue deposition, as an early stage of ECM mineralization (ectopic bone tissue formation) occurred from the periphery to the central region of the scaffolds. Even in implants into bone critical defects (4 mm), 4 weeks should not be enough time to observe mature bone tissue. Usually, in ectopic bone formation model, the bone tissue growth should be observed only after 12 weeks (Scotti et al., 2013). In a previous work, no evidence of the material's bovine collagen was observed in a study after 8 weeks post-implantation, suggesting that scaffold biodegradation by endogenous collagenase had taken place, without impairing tissue regeneration (Salgado et al., 2016). These findings suggest an enhancement of human MSC's activity, as would be expected with the molecular stimuli present on the materials' surface (OPS). Since the scaffolds composition and cellular organization of the pre-formed cell culture undergo dynamic changes after implantation, it was important to evaluate the materials tissue engineering potential application. Therefore, to track the implanted HBMSCs, a specific human nucleic marker was used (HuNu, nuclear pink-stain) (Figure 9). Human cell culture presence in the scaffold and migration capacity was observed from the early times of implantation (7 days) to the latest ones (28 days). These cells were viable as they secreted human OPN (natural bone ECM), these findings are in accordance with the number of HBMSC's as the protein presence is more evident in the materials with OPS modification. OPS signaling toward the cells cultured into the scaffolds probably were responsible for the bone differentiation of the HBMSC in the ectopic bone formation on the mice model and also allowed the transplanted human cells not only to survive, but to grow, migrate and differentiate into the desired tissue.

Altogether, our findings support the bone regenerative potential of the biocomposite scaffold modified with phosphoserine and potentially regulate the cellular bone differentiation after the scaffold implantation. Upon the explant evaluation, high cellular proliferation of subcutaneous tissue was observed, with more organized conjunctive tissue and full-filling all the 3D material structure. Also, with the human MSC's, phosphoserine signaling promoted higher tissue mineralization in comparison to the control group (only collagen and nanohydroxyapatite). In the future, new studies are required to acquire a comprehensive understanding of how OPS presence supports bone healing and its effect on cell migration and differentiation.

## Conclusions

The main advantage of scaffold modification with O-phospho-L-serine by cryogel method is the osteoinductive signaling, without biocompatible manufacturing. Moreover, the hydroxyapatite nanoparticles have significant influence on the materials behavior, but slight modifications could be observed on the materials mechanical properties with the OPS presence. The *in vitro* cell behavior analysis with MG63 and HBMSC cells exhibited a progressively strong cell viability and proliferation along the period of observation. After these *in vitro* results, the ideal composition was identified as being 2.5% of phosphoserine (Coll/nanoHA_OPS5) by detecting similar values either for ALP enzyme activity or for several differentiation gene markers through qPCR analysis (OC, Runx-2 OPN) of the positive control (osteoinduction supplement). Ectopic bone formation animal model results of materials loaded with HBMSCs showed increased calcium tissue deposition in the presence of phosphoserine. Also, lower implantation periods showed a reduced inflammatory response with new micro-vascularization formation inside and around the implant. The data from this work indicate that the biocomposite with higher content of O-phospho-L-serine had higher capacity to promote differentiation of human MSC's, enhance cell viability and bone tissue ECM production (OPN and presence of calcium ions) *in vivo*. The OPS signaling composite scaffold showed an appropriated design, cell distribution and behavior that provide characteristics similar to those of normal bone tissue favoring its application in bone tissue engineering.

## Data Availability

All datasets generated for this study are included in the manuscript/Supplementary Files.

## Ethics Statement

The animal study was reviewed and approved by Ethical Animal Commission of i3S, Portugal.

## Author Contributions

CS contributed substantially to the conception and design of the biocomposite, biological and animal experiments, performed most acquisition, analysis, and interpretation of data. CS agreed to be accountable for all aspects of the work in ensuring that questions related to the accuracy or integrity of any part of the work are appropriately investigated and resolved. CS prepared and revised the draft critically for important intellectual content. BT contributed to the production of the biocomposite and performed parts of the physicochemical analysis, revised parts of the draft, and give final approval of the version to be published. FM critically revised the draft for important intellectual content and give final approval of the version to be published.

### Conflict of Interest Statement

The authors declare that the research was conducted in the absence of any commercial or financial relationships that could be construed as a potential conflict of interest.
