# Supplementary material for: Biomimetic Composite Scaffold With Phosphoserine Signaling for Bone Tissue Engineering Application
Source: Front Bioeng Biotechnol. 2019 Sep 6;7:206. doi: 10.3389/fbioe.2019.00206 (PMC6743420; doi:10.3389/fbioe.2019.00206)
Supplement: Supplementary file 1 [file Table_1.docx]

Supplementary Material

# Supplementary Data

# O-Phospho-L-Serine cellular biocompatibility assay

For the *in vitro* study, a human primary MSC’s (Bone marrow stromal cells – HBMSC, Hospital São João, Portugal) were cultured until the third passage (P3) and the biocompatibility of the samples was evaluated using Alamar blue assay (Rezasurin, Sigma-Aldrich). Cells were seeded into 24-well plates at 5x10^3^ cells per well. After 24 h, complete medium with 43.75 to 350 mg/mL of OPS powder. The medium was changed each 3 days. After the time points, the supernatant was removed and a solution with rezasurin (0.1 mg mL^-1^, Sigma-Aldrich) was incubated with the cell culture for 3 hours. Then, the fluorescence intensity of the supernatant was measured with a fluorimeter (Synergy Mx, BioTek). As control, cells cultured in polystyrene well –plates (TCPS) were evaluated after the same time-points. In all time-points, cellular culture of primary human bone marrow stromal cells (HBMSC) had similar viability to the control with or without the osteogenic induction (Supp.Fig. 1).

**Supplementary figure 1:** Cell viability of HBMSC cells cultured with phosphoserine (OPS – 350 - 43 μg/mL) for 7, 14 and 21 days.

The alkaline phosphatase activity was measured as quantitative analysis for early osteogenic characterization. HBMSC were cultured in the presence of different concentrations of OPS (43.75-350 mg/mL) and after 3 weeks, cells were detached from the plates and lysed for 30 min with 1 % Triton X-100 solution (Sigma-Aldrich). The enzyme activity was measured as described in the work and the results were normalized to total protein content (nanomoles per minute per microgram of protein – showed at Supp. Fig. 4).

Higher ALP activity was observed with the highest concentration of phosphoserine after 14 days with or without the osteogenic medium (Supp.Fig. 2). Thus, the presence of this phosphorylated amino acid probably induces the differentiation.

**Supplementary figure 2:** ALP activity for osteoblastic phenotype expression of human bone marrow stromal cells (HBMSC) cultured with phosphoserine (OPS) at different concentrations (350-86 μg/mL) for different time points (7-21 days). Differences between cells with and without OPS (control - TCPS) were statistically significant (p < 0.05).

*****

*****

*****

*****

Total RNA was extracted from cell cultures on the scaffold material using the NucleoSpin (Macherey-Nagel) kit according to the manufacturer’s instructions and subjected to reverse transcriptase polymerase chain reaction (RT-PCR) amplification (Titan One Tube RT-PCR system; Roche, Branchburg, NJ) for 30 cycles. RT reaction mixtures consisted of extracted RNA, Titan RT-PCR buffer, dithiothreitol, deoxynucleoside triphosphate, primers for each gene tested, avian myeloblastosis virus RT (AMV-RT), and water in a total volume of 25 µL. Total RNA was reverse transcribed with AMV-RT, at 60 ^o^C for 30 min, followed by a 2 minutes denaturation at 94 ^o^C. The complementary DNAs (cDNAs) were then amplified with recombinant Taq-DNA polymerase under the following conditions: 30 cycles of denaturation (94 ^o^C/30 s), annealing (55 ^o^C/30 s), elongation (68 ^o^C/45 s), followed by a prolonged elongation of 7 min at 68 ^o^C. Table 1 shows the primer sequences used for PCR amplification. To obtain a semi-quantitative assessment of gene expression, data were expressed as normalized ratios, by comparing the integrated density values for all tested genes with those for glyceraldehyde-3-phosphate dehydrogenase (GAPDH). The PCR products were separated by 1% agarose gel electrophoresis and visualized by GelRed™ Nucleic Acid Stain (Biotium). The images of the gel were captured with a camera and analyzed with Image J software.

OPG is a soluble receptor of RANK and is synthesized by mature osteoblasts. Studies in animal models have indicated that the major biological effect of OPG is to inhibit osteoclast differentiation and activity. The optimal concentration of OPS leaded to higher expression for osteogenic genes such as OPG, Runx-2 and OC in human pre-osteoblast cell line (MG63) after 2 weeks and human MSC’s (HBMSC) after 1 week measured through RT-PCR analysis.

**Supplementary figure 3:** RT-PCR expression cellular results after cultured on Coll/nanoHA (50:50 wt%) with or without OPS5 for 7 and 14 days (A - MG63 cells and B - HBMSCs).

Cell differentiation was more evident on the scaffolds containing OPS, after 14 days of the MG63 cell culture, which could be related to the osteoconductive element released during the *in vitro* culture (Supp.Fig. 3A and B).

**Supplementary figure 4:** Total protein content (Lowry’s method) of scaffolds with and without OPS signaling after 7, 14 and 21 days of HBMSC’s culture.
