# Supplementary figures and images for: Biomimetic Composite Scaffold With Phosphoserine Signaling for Bone Tissue Engineering Application
Source: Front Bioeng Biotechnol. 2019 Sep 6;7:206. doi: 10.3389/fbioe.2019.00206 (PMC6743420; doi:10.3389/fbioe.2019.00206)

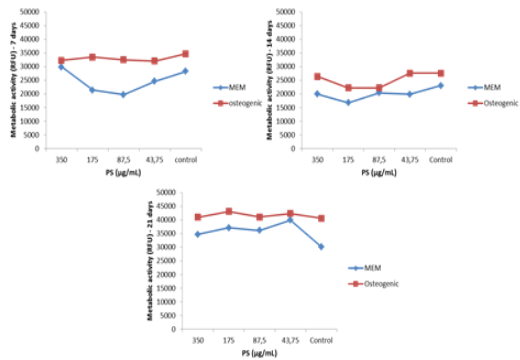

Supplement: Supplementary file 2 [file Image_1.TIFF]

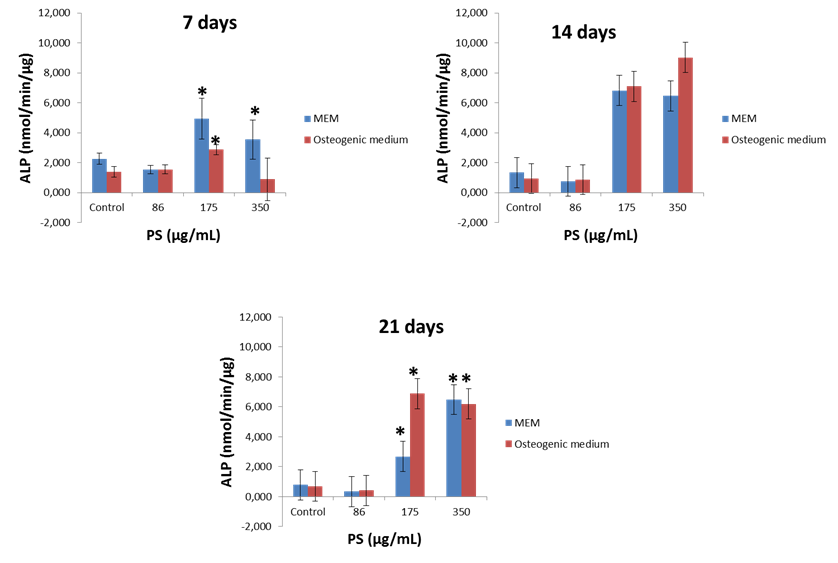

Supplement: Supplementary file 3 [file Image_2.TIFF]

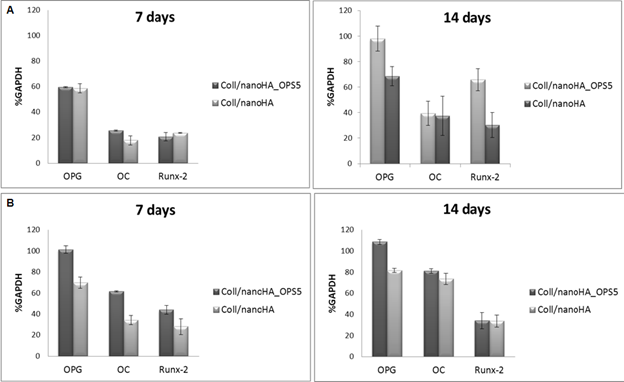

Supplement: Supplementary file 4 [file Image_3.TIFF]

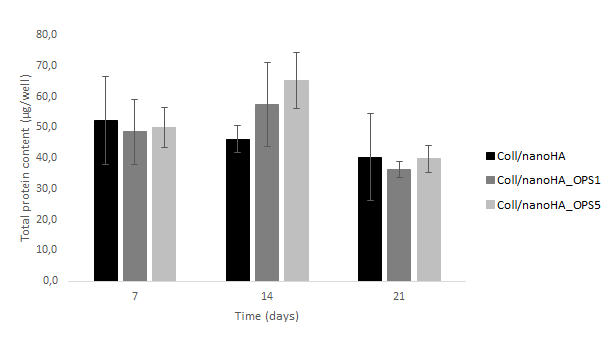

Supplement: Supplementary file 5 [file Image_4.TIFF]
